# Membrane-bound full-length Sonic Hedgehog identifies cancer stem cells in human non-small cell lung cancer

**DOI:** 10.18632/oncotarget.21781

**Published:** 2017-10-10

**Authors:** Etienne Giroux Leprieur, Bhairavi Tolani, Hui Li, Fleur Leguay, Ngoc T. Hoang, Luis A. Acevedo, Joy Q. Jin, Hsin-Hui Tseng, Dongsheng Yue, Il-Jin Kim, Marie Wislez, Changli Wang, David M. Jablons, Biao He

**Affiliations:** ^1^ Thoracic Oncology Program, Department of Surgery, Helen Diller Family Comprehensive Cancer Center, University of California, San Francisco, CA, USA; ^2^ Department of Respiratory Diseases and Thoracic Oncology, APHP - Ambroise Pare Hospital, Boulogne-Billancourt, France; ^3^ EA 4340, UVSQ, Paris-Saclay University, Boulogne-Billancourt, France; ^4^ Department of Lung Cancer, Lung Cancer Center, Tianjin Medical University Cancer Institute and Hospital, Tianjin, China; ^5^ Sorbonne University, UPMC GRC-04 Theranoscan, Department of Respiratory Diseases, APHP - Tenon Hospital, Paris, France

**Keywords:** non-small cell lung cancer (NSCLC), Sonic Hedgehog (Shh), cancer stem cells (CSCs), GDC0449, chemo-resistance

## Abstract

The mechanism of Sonic Hedgehog (Shh) pathway activation in non-small cell lung cancer (NSCLC) is poorly described. Using an antibody against the Shh C-terminal domain, we found a small population of Shh-positive (Shh+) cells in NSCLC cells. The objective of this study was to characterize these Shh+ cells. Shh+ and Shh- cells were sorted by using Fluorescence Activated Cell Sorting (FACS) on 12 commercial NSCLC cell lines. Functional analyses on sorted cells were performed with gene expression assays (qRT-PCR and microarray) and cells were treated with cytotoxic chemotherapy and a targeted inhibitor of Shh signaling (GDC0449). We used *in vivo* models of nude mice inoculated with Shh+ and Shh- sorted cells and drug-treated cells. Finally, we confirmed our results in fresh human NSCLC samples (n=48) paired with normal lung tissue. We found that Shh+ cells produced an uncleaved, full-length Shh protein detected on the membranes of these cells. Shh+ cells exerted a paracrine effect on Shh- cells, inducing their proliferation and migration. Shh+ cells were chemo-resistant and showed features of cancer stem cells (CSCs) *in vitro* and *in vivo*. Pharmacological inhibition of the Shh pathway suppressed their CSC features. A high percentage of Shh+ cells was associated with poor prognosis in early-stage NSCLC patients. In conclusion, we describe for the first time the presence of an abnormal membrane-bound full-length Shh protein in human cancer cells that allows the identification of CSCs *in vitro* and *in vivo*.

## INTRODUCTION

Lung cancer is the main cause of cancer-related mortality in the world, and non-small cell lung cancer (NSCLC) is the predominant histological subtype [1]. Since the majority of advanced NSCLC does not present a targetable oncogenic signature, systemic chemotherapy plays a major treatment role for most of these malignancies. However, the benefits of chemotherapy are modest. Therefore, there is an urgent need to develop novel therapies based on understanding the molecular mechanisms of chemo-resistance.

Sonic Hedgehog (Shh) is a protein that is physiologically involved in vertebrate development [2–4]. The full-length protein is cleaved in the cytosol into N- and C-terminal products [5, 6]. Whereas the C-terminal peptide is freely secreted, the N-terminal peptide is modified by lipid hydrophobic modifications and retained in the membrane before its secretion [5–7]. During development, only the N-terminal product has biological activity [4, 8–12]. A reactivation of the Shh pathway has been described in a variety of malignancies [13], including NSCLC [14, 15]. This pathway is involved in carcinogenesis of various tumors [16–19] and seems to play a role in cancer stem cell (CSC) maintenance [20–22]. However, the role of the Shh pathway in NSCLC is poorly described.

Here we investigated the role of the Shh pathway in NSCLC. We show that a small proportion of cells produce the full-length Shh protein (Shh+ cells). This subpopulation has CSC features, is the “signal source” for maintaining survival of NSCLC cells through paracrine mechanisms, and is the driver for drug resistance.

## RESULTS

### Presence of NSCLC Shh+ cells *in vitro*

**Figure 1:**
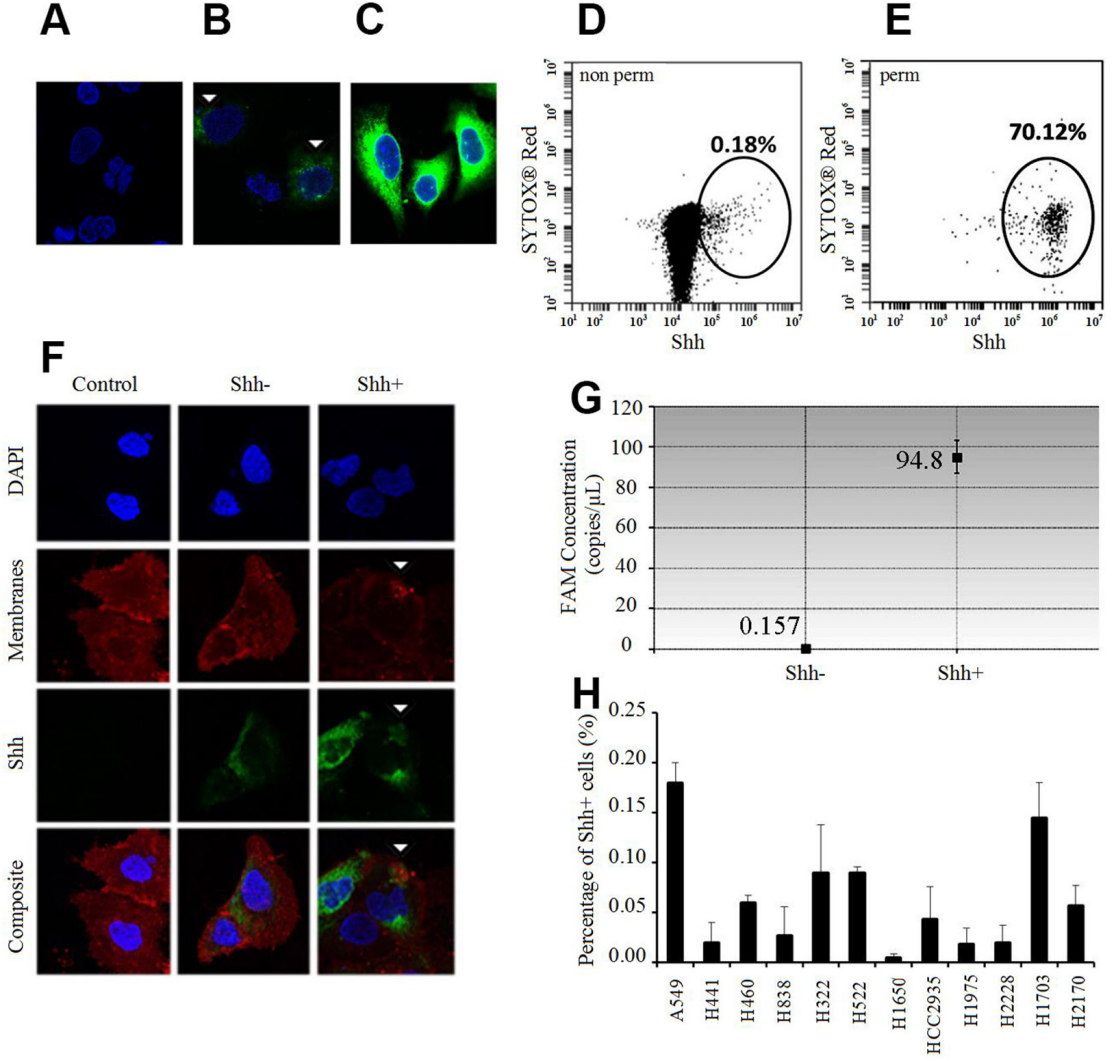
Shh+ cells produce Shh and are rare *in vitro* **(A-C)** Immunofluorescence (IF) analysis of A549 cells relative to the unstained controls (A: only secondary antibody) without membrane permeabilization (B) shows positive Shh (green) and nuclear staining (blue, DAPI). White arrows: positive membranous Shh staining in relatively few cells. (C) IF analysis of A549 cells with membrane permeabilization (Triton X-100) shows positive Shh (green) and nuclear staining (blue, DAPI) in a majority of the cells probed. **(D)** Flow cytometric analysis of A549 cells without membrane permeabilization probed for Shh (0.18%). **(E)** Flow cytometric analysis of A549 cells with membrane permeabilization (Tween 20) probed for Shh (70.12%). **(F)** IF analysis of sorted A549 cells without membrane permeabilization shows strong positive membranous Shh staining (green, white arrows) in Shh+ cells and low/no staining in Shh- cells/controls without the primary antibody. Red: membranous staining (lipophilic dye); blue: nuclear staining (DAPI). **(G)** *Shh* gene expression analysis by ddPCR in A549 Shh+ and Shh- cells. **(H)** Percentage of Shh+ cells (%, mean ±SD) in several NSCLC cell lines.

We developed a peptide antibody against the C-terminal of Shh and tested its specificity with and without permeabilization on NSCLC cells. Surprisingly, immunofluorescence (IF) analysis showed that relative to unstained cells (Figure 1A, secondary antibody only), only a very small number of cells stained positive (Shh+) without permeabilization (Figure 1B); with permeabilization, most were positive (Figure 1C). Flow cytometry analysis corroborated these findings in showing that less than 0.20% (Figure 1D) and more than 70% (Figure 1E) of the cells were positive without and with permeabilization respectively. To better characterize these Shh+ cells by IF and flow cytometry, we sorted them via Fluorescence Activated Cell Sorting (FACS) without membrane permeabilization. We confirmed by IF analysis on sorted cells that only Shh+ cells had a Shh membranous staining, whereas Shh- cells had low or no staining (Figure 1F). We then showed that Shh+ cells produced Shh by digital droplet PCR (ddPCR) analysis in sorted cells (Shh+ and Shh- cells). We observed that Shh+ cells expressed high levels of the *Shh* gene [FAM concentration=94.8 copies/μl (Poisson confidence interval: 87.1-103)], whereas Shh- cells did not express the *Shh* gene [FAM concentration=0.157 copies/μl (Poisson confidence interval: 0.01-0.75)] (Figure 1G). Next, we screened 12 NSCLC cell lines (10 adenocarcinoma cell lines: A549, H322, H441, H460, H522, H838, H1650, H1975, H2228, HCC2935; 2 squamous cell lines: H1703, H2170) by flow cytometry on non-permeabilized cells. We found that 0.06% (± 0.05%) of the cells were Shh+ via flow cytometry analysis (Figure 1H). The highest Shh+ percentage was for A549 at 0.18% (± 0.02%).

### Secretion of full-length Shh protein by Shh+ cells

**Figure 2:**
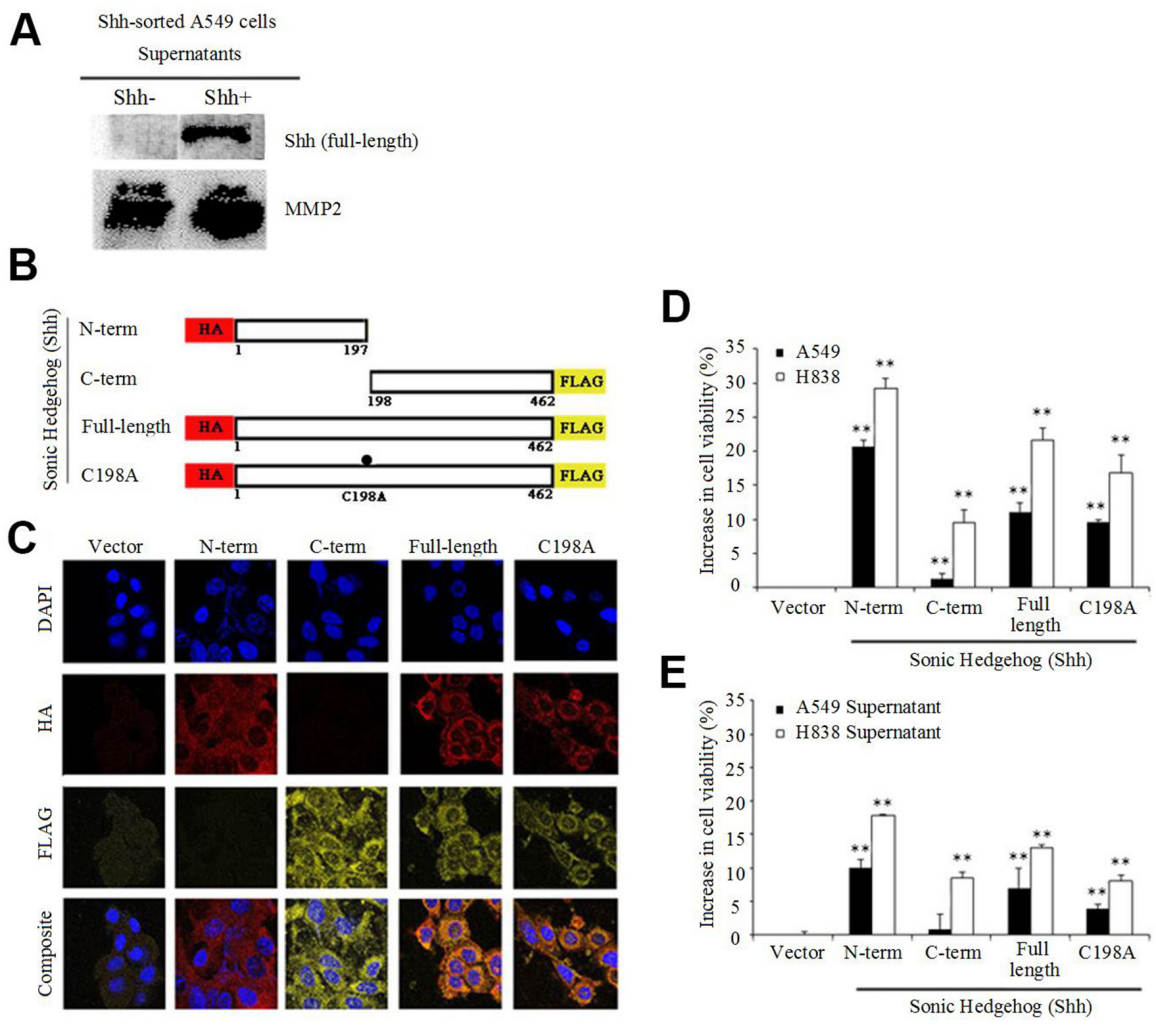
Shh+ cells produce Shh full-length protein **(A)** Immunoblot of supernatants from non-transfected Shh-sorted A549 cells probed for the Sonic Hedgehog (Shh) protein, with secreted MMP2 as a loading control. **(B)** Schematic representation of Shh constructs showing the sizes and locations of N-term HA and C-term FLAG tags stably expressed in NSCLC cells. **(C)** Immunofluorescence analysis of H838 cells showing cytosolic and membrane staining of N-term, C-term, wild-type Shh and C198A Shh constructs probed for the presence of HA (red) and FLAG (yellow). **(D)** NSCLC cell lines (A549 and H838) used in (C), analyzed for increases in viability (MTS assay) relative to the vector control after 4 days (^**^p<0.01). **(E)** Supernatants from NSCLC cells were applied to parental cells and analyzed as in D (^**^p<0.01).

To characterize the localization of the Shh protein recognized by the C-terminal Shh antibody, we used NSCLC cell line A549 transiently transfected with the *Shh* gene. Since we used a C-terminal specific antibody, our hypothesis was that we would identify either the C-terminal Shh peptide, or the Shh full-length protein on the cell surface. Western blotting (WB) indicated the presence of the full-length Shh protein, both in the cytosol and on the membrane (Supplementary Figure 1) recognized by the C-term Shh antibody. Moreover, WB analysis on the culture medium (supernatant) of non-transfected A549 sorted cells (3 days after sorting) showed the presence of the full-length Shh protein in the supernatant only for Shh+ cells, but not for Shh- cells, indicating that Shh- cells did not secrete the protein (MMP2 served as a loading control) (Figure 2A). Most published studies describe the secretion and the activity of the N-terminal Shh peptide, notably during development [4, 8–12]. To further characterize the localization and functional significance of the Shh protein and its cleaved products, we used retrovirus-mediated gene transfer to stably express several versions of the *Shh* gene in A549 and H838 cells. We used N-term peptide hemagglutinin (HA)-tagged Shh (1-197aa), C-term peptide FLAG-tagged Shh (198-462aa), double-tagged wild-type Shh (N-HA and C-FLAG) and double-tagged cleavage mutant Shh C198A (N-HA and C-FLAG) as shown in Figure 2B. The presence of the C198A mutation is known to induce the production of a processing-defective full-length Shh protein [23]. Next, we confirmed peptide expression and membrane/cytosolic localization of N-term, C-term, wild-type and C198A mutant Shh via immunofluorescence in both A549 (Supplementary Figure 2A) and H838 cells (Figure 2C) with antibodies directed at HA and FLAG respectively. Flow analysis revealed positive double staining in H838 cells for HA and FLAG in cells bearing wild-type and C198A constructs, and single staining for N-term and C-term (Supplementary Figure 2B). Functional analyses with stably expressing N-term Shh in A549 and H838 cells resulted in a 20-30% growth advantage compared with the vector control (Figure 2D), consistent with the role of N-term Shh in biological development [4, 8–12]. Moreover, the wild-type and C198A-expressing lines also showed significant increases in viability (10-20% and 10-15%, respectively) relative to the vector. The C-term expressing lines only showed a 1-10% increase. We observed analogous results when we applied the supernatants from cells expressing either vector, N-term, C-term, wild-type, or C198A to parental cell lines and we report a 20% increase in the viability for N-term relative to the vector control (Figure 2E).

### Paracrine effect of Shh+ cells on other cancer cells

**Figure 3:**
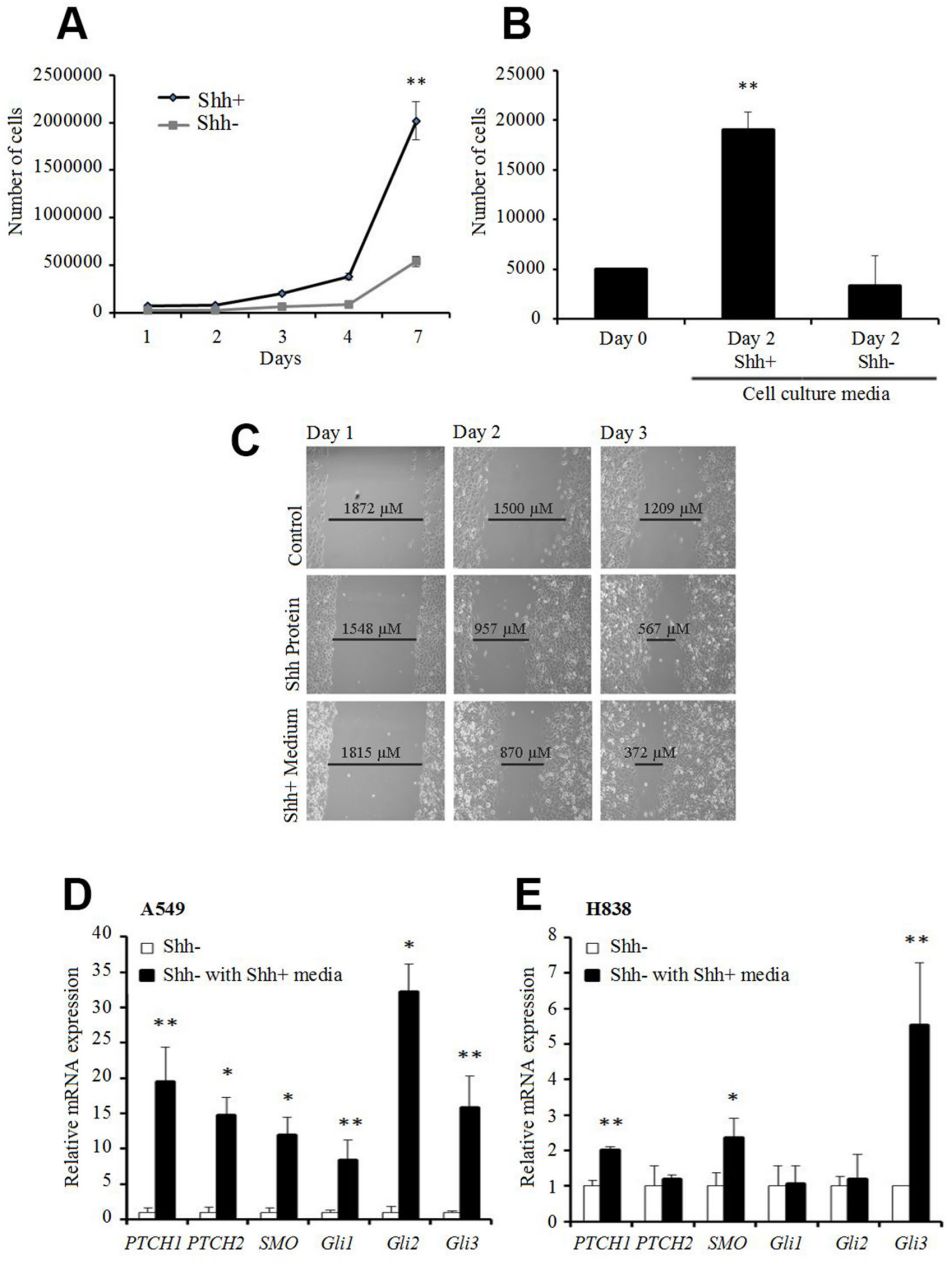
Shh+ cells have a paracrine effect on Shh- cells **(A)** Proliferation assay (MTS) on A549 Shh+ and Shh- cells [d0, d3, d4, d7 (^**^p<0.01)]. **(B)** proliferation assay (MTS) of A549 Shh- cells supplemented with culture media from Shh+ or Shh- cells [conditioned media: fresh media (1:1) at d1 after cell sorting]. ^**^p<0.01, compared with cells supplemented with media from Shh-cells. **(C)** migration/wound healing assay on A549 Shh- cells at day 1 (left), day 2 (middle) and day 3 (right) (top to bottom: control, 1,200 ng/mL Shh recombinant protein, medium from Shh+ cells). Representative images from three independent experiments are presented. **(D** and **E)** gene expression levels of downstream Shh pathway targets analyzed by qRT-PCR in A549 (D) or H838 (E) Shh- cells cultured with media from Shh+ cells (normalized to Shh- cells). ^*^p<0.05; ^**^p<0.01, compared with Shh- cells cultured with media from Shh- cells.

To better understand the properties of Shh+ and Shh- populations, we performed functional analyses on sorted cells. We noted that the total number of cells after 7 days in culture was 4 times higher for Shh+ cells than for the Shh- cells (Figure 3A). Moreover, the addition of media from Shh+ cells to Shh- cells induced a higher proliferation rate and increased the migration rate of the Shh- cells by 50%. Addition of human recombinant N-terminal Shh peptide increased the migration rate by 15% (Figure 3B & 3C). We also showed that the addition of media from Shh+ cells to Shh- cells induced gene overexpression of the downstream components of the Shh pathway such as *Ptch*, *Smo*, *Gli1, Gli2*, and *Gli3* in quantitative RT-PCR (qRT-PCR) (Figure 3D & 3E). Taken together, these results suggest a paracrine role of Shh+ cells on Shh- cells, with activation of downstream factors of the Shh pathway putatively resulting in tumor proliferation and aggressiveness.

### Effect on the percentage of Shh+ cells following cisplatin, docetaxel and GDC0449 treatment

**Figure 4:**
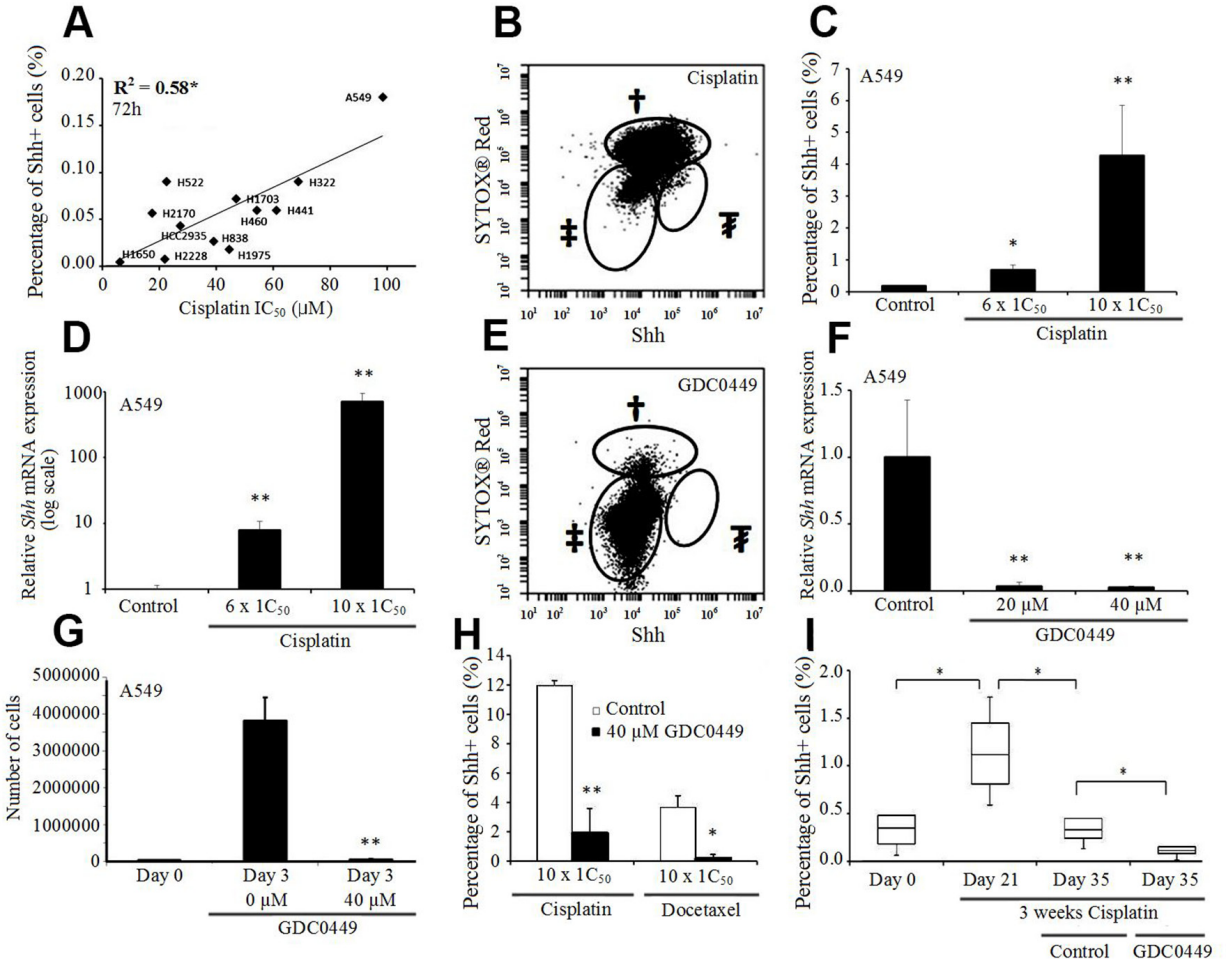
Shh+ cells are resistant to cisplatin but sensitive to GDC0449 **(A)** Correlation between the percentage of Shh+ cells (%) and cisplatin IC_50_ in NSCLC cell lines. **(B)** Percentage of Shh+ cells (%) in A549 cells treated with cisplatin (1 mM, 72h). †dead cells; ‡Shh- cells; ₮Shh+ cells (5% of live cells). **(C)** Percentage of Shh+ cells (%) in A549 cells treated by cisplatin (600 μM and 1 mM, 72h). ^*^p<0.05; ^**^p<0.01, compared to baseline. **(D)** *Shh* expression level (qRT-PCR) in A549 cells treated with cisplatin (600 μM and 1 mM, 72h) (normalized to PBS-treated cells, log-scale). ^**^p<0.01, compared to PBS. **(E)** Percentage of Shh+ cells (%) in A549 cells treated with GDC0449 (40 μM, 72h). †dead cells; ‡Shh- cells; ₮Shh+ cells (0%). **(F)** *Shh* expression level (qRT-PCR) in A549 cells treated with GDC0449 (20 μM and 40 μM, 72h) (normalized to DMSO-treated cells). ^**^p<0.01, compared to DMSO. **(G)** MTS assay of A549 cells treated with DSMO or GDC0449 (40 μM, 72h). ^**^p<0.01, compared to day 0. **(H)** Percentage of Shh+ cells (%) in A549 cells treated with cisplatin and DMSO/GDC0449 (40 μM, 72h) or docetaxel and DMSO/GDC0449 (40 μM, 72h). ^**^p<0.01, compared to cisplatin/docetaxel and DMSO. **(I)** Percentage of Shh+ cells (%) in A549 xenograft model treated with cisplatin (CPT, 10 mg/kg, IV weekly), followed by vehicle or GDC0449 (20 mg/kg, IP daily). ^*^p<0.05.

To examine the correlation between the cisplatin sensitivity and the percentage of Shh+ cells, we compared the resistance to cisplatin with the level of Shh expression and observed a positive correlation between the IC_50_ of cisplatin and the percentage of Shh+ cells assessed by flow cytometry (p=0.004, R^2^=0.58, Figure 4A). Furthermore, when we treated the cells with escalating doses of cisplatin, we saw a corresponding augmentation in the percentage of Shh+ cells (Figure 4B & 4C; Supplementary Figure 3A-3C), and an increase in the total number of Shh+ cells. We further confirmed this elevation in *Shh* gene expression by qRT-PCR after cisplatin treatment (Figure 4D; Supplementary Figure 3D-3F).

Treatment of A549 cells with 40 μM GDC0449 (vismodegib/commercial Smoothened inhibitor) successfully inhibited the Shh pathway, as evidenced by a complete disappearance of Shh+ cells assessed by flow cytometry (Figure 4E). qRT-PCR analyses confirmed this dramatic decrease in *Shh* gene expression after GDC0449 treatment (Figure 4F; Supplementary Figure 4). In addition, treatment with GDC0449 induced an inhibition of cell proliferation and migration (Figure 4G; Supplementary Figure 5). Moreover, we observed that when GDC0449 was combined with chemotherapy (cisplatin or docetaxel), the percentage of Shh+ cells decreased significantly compared to chemotherapy alone (Figure 4H). To confirm these results *in vivo*, we used A549 xenografts in nude mice treated sequentially with cisplatin and then GDC0449, or treated with the combination of docetaxel and GDC0449. We observed a significant increase in the percentage of Shh+ cells after chemotherapy, a significant reduction in the percentage of Shh+ cells after GDC0449 treatment (sequential assay), and a significant reduction in the percentage of Shh+ cells following combination treatment, compared to chemotherapy alone (Figure 4I; Supplementary Figure 6).

### CSC features of Shh+ cells

To elucidate the features of the two cell populations, we performed gene expression microarray profiling of sorted Shh+ and Shh- A549 cells (Supplementary Figure 7). This analysis revealed an overexpression of Shh pathway genes (notably *Shh*), several well-known CSC genes (*POU5F1P3, NANOG, LIN28A, ALDH1A2, PROM1*), genes involved in cell survival and proliferation [Cyclin D2 (*CCND2*), *BCL2*], Wnt pathway genes, and chemokine-related genes. Interestingly, genes associated with differentiation (*KRT7, PRDM1*) were under-expressed in Shh+ cells.

**Figure 5:**
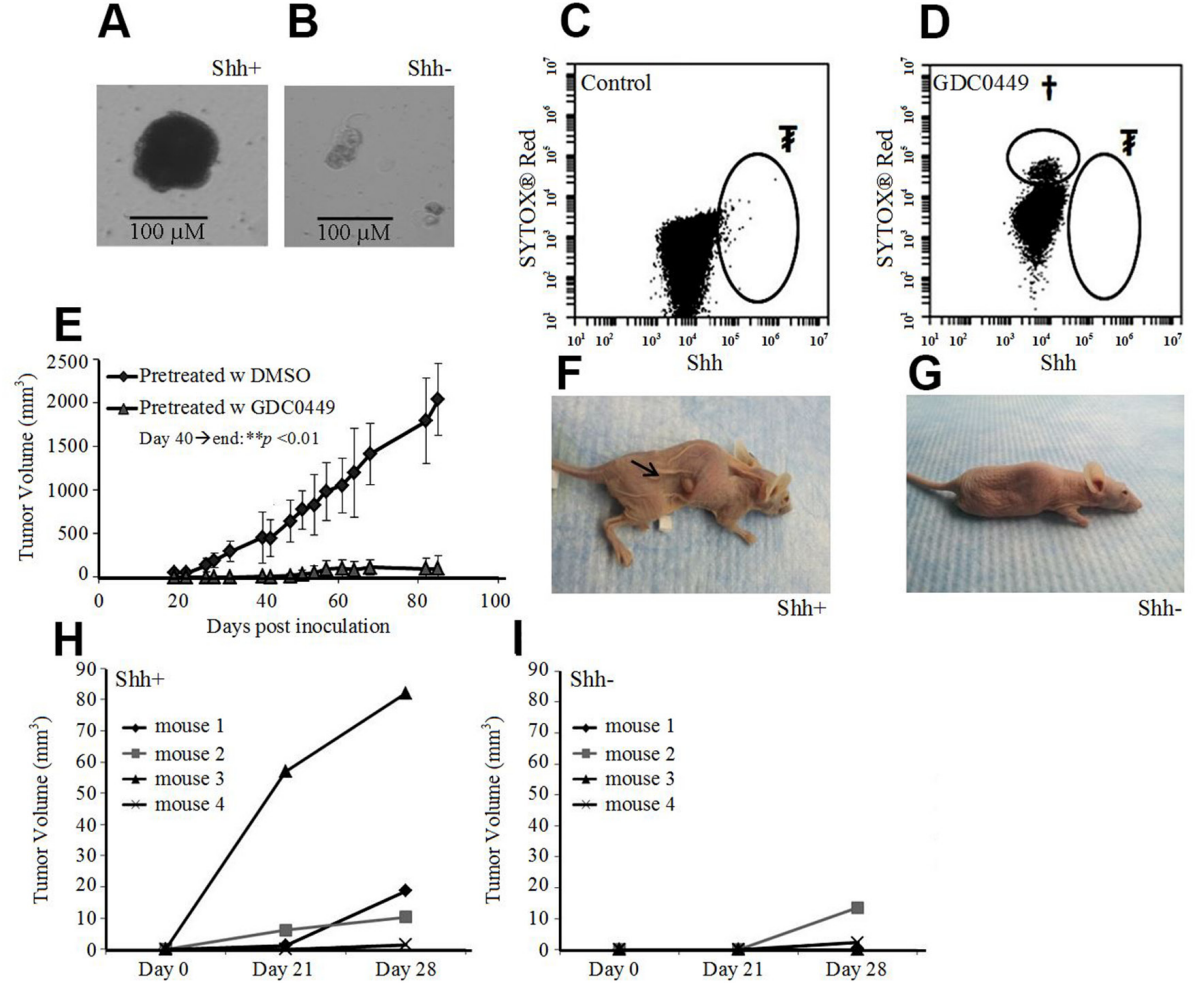
Shh+ cells have features of cancer stem cells **(A)** Growth of A549 Shh+ cells cultured under serum-free conditions at d14 led to spheroid formation (scale bar: 100 μm). **(B)** culture of A549 Shh- cells in serum-free media at d14 did not lead to spheroid formation (scale bar: 100 μm). **(C)** flow cytometric analysis of pre-inoculated A549 cells treated with DMSO (d3). ₮Shh+ cells (0.10%). **(D)** flow cytometric analysis of pre-inoculated A549 cells treated with GDC0449 (40 μM, d3), †dead cells (SytoxRed); ₮Shh+ cells (0%). **(E)** Change in A549 xenograft tumor volume in nude mice after inoculation of pre-treated cells (d40 until the end of follow up: ^**^p<0.01). **(F)** Nude mice inoculated with a low amount (1,500) of A549 Shh+ cells with tumor formation at 3 weeks. **(G)** nude mice inoculated with a low amount (1,500) of A549 Shh- cells, without tumor formation at 3 weeks. **(H** and **I)** subcutaneous tumor volumes in nude mice 3-4 weeks after inoculation of a low amount (1,500) of A549 Shh+ (H) or Shh- cells (I).

When sorted cells were cultured in serum-free media, floating spheroids with A549 Shh+ cells formed 10 to 15 days after sorting (Figure 5A), but not with A549 Shh- cells (Figure 5B). To prove the critical role of the Shh pathway in oncogenesis, we treated cultured A549 cells with 40 μM of GDC0449 (Figure 5C & 5D). Then, we inoculated nude mice subcutaneously with 1 million treated cells (still adherent to the culture flask after treatment with GDC0449). We observed that tumor formation was completely inhibited with the pre-treated GDC0449 cells compared with the control group (DMSO pre-treated cells) (Figure 5E). Next, we inoculated nude mice with a small number (1,500 cells) of Shh+ or Shh- cells subcutaneously. At 3 weeks, 3 out of 4 mice (75%) inoculated with Shh+ cells developed a measurable xenograft, whereas 0 of 4 mice (0%) inoculated with Shh- cells developed any tumors (Figure 5F-5I). These data suggest that Shh+ cells are CSCs and that the Shh pathway is a key component in oncogenesis.

### Presence of Shh+ cells in human tumor samples and prognostic impact

**Figure 6:**
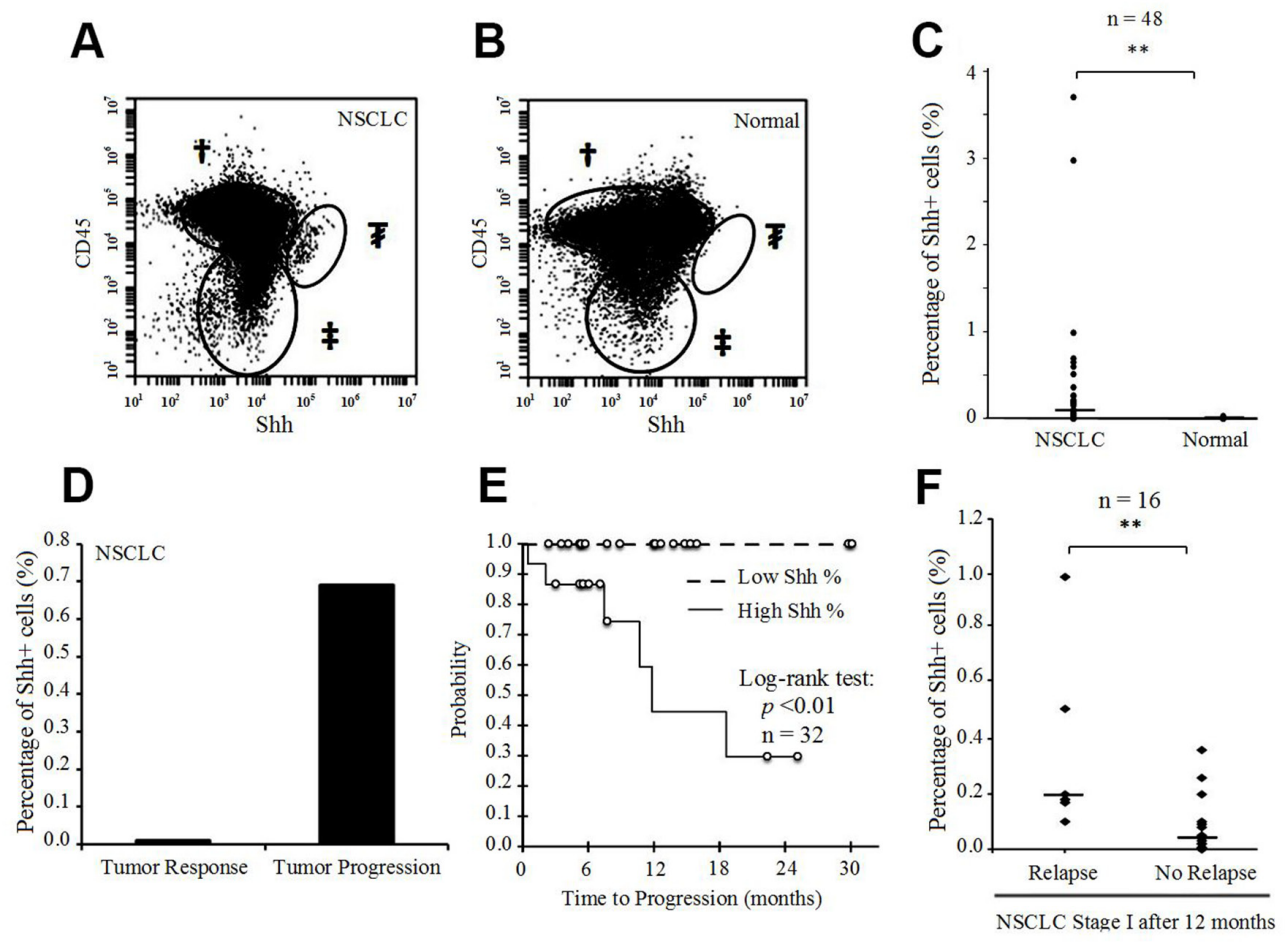
Presence of Shh+ cells in fresh human NSCLC tissue samples **(A)** Flow cytometric analysis of Shh in a fresh human NSCLC sample [†CD45+ cells; ‡CD45-Shh- cells; ₮CD45-Shh+ cells (2.99% of CD45- cells)]. **(B)** Flow cytometric analysis of Shh in a fresh human normal lung sample [†CD45+ cells; ‡CD45-Shh- cells; ₮CD45-Shh+ cells (0%)]. **(C)** Percentage of Shh+ cells (%) in fresh human NSCLC samples and corresponding normal lung tissues (n=48). **(D)** Percentage of Shh+ cells (%) in primary lung adenocarcinoma with a tumor response after chemotherapy and the corresponding adrenal metastasis with tumor progression after chemotherapy in the same patient. **(E)** Time-to-progression (TTP) according to high (>0.10%) or low (<0.10%) percentage of Shh+ cells (p<0.01 for log-rank test) in stage I NSCLC tissue samples (n=32). **(F)** Percentage of Shh+ cells (%) in stage I NSCLC with tumor relapse <12 months after surgery (n=6) and in stage I NSCLC without relapse within 12 months after surgery (n=16) (^**^p<0.01).

To evaluate the role of Shh as a prognostic marker in lung cancer, we tested 48 fresh human surgical samples from 47 patients with NSCLC, obtained consecutively from the Surgery Department at UCSF. There were 46 primary lung tumors, one tumor pleural effusion, and one adrenal metastasis of lung adenocarcinoma. Patient samples were probed for Shh to assess the percentage of positive cells, and for CD45 to exclude immune cells from the final analysis. Although the overall median of percentage Shh+ cells was 0.06% (interquartile range IQR 0.02-0.20), the percentage in corresponding fresh normal lung tissue (n=48) was 0% (IQR 0-0%) (p<0.001) (Figure 6A-6C). The median percentage of Shh+ cells was 0.09% (IQR 0.03-0.21) for stage I (n=32), 0.02% (IQR 0.01-0.11) for stage II (n=6), 0.01% (IQR 0-0.02) for stage III (n=5) and 0.15% (IQR 0.01-0.56) for stage IV (n=5) (p>0.05 for each comparison). All the samples were chemo-naive, except for 3 samples (stage I: n=1; stage IV: n=2) from patients who underwent chemotherapy before surgery. One patient underwent surgery twice after systemic chemotherapy, with 6-weeks delay between the 2 surgeries (the first surgery for the primary lung tumor; the second for an adrenal metastasis), and without any chemotherapy between the 2 surgeries. Initial post-chemotherapy evaluation showed a tumor response in the primary lung tumor, but a tumor burden on the adrenal metastasis. Flow cytometry analysis showed a nearly 70-fold higher percentage of Shh+ cells at the metastatic progressive site than in the primary tumor that responded to chemotherapy (Figure 6D). qRT-PCR analysis confirmed this result of *Shh* gene overexpression at the site with tumor progression compared to the site with tumor response (p=0.01, Supplementary Figure 8). We also tested fresh lung metastases from other pathological subtypes via flow cytometry and observed the presence of Shh+ cells in almost all of them (Supplementary Table 1). We then performed microarray analysis on 7 sorted fresh human tumor samples {4 primary lung adenocarcinoma and 3 secondary lung tumors [primary: melanoma (n=1); breast carcinoma (n=1); colorectal carcinoma (n=1)]}. Consistent with our microarray data from the NSCLC cell line, we found gene overexpression for Shh pathway genes, CSC genes, cell survival, EMT and aggressiveness genes, Wnt pathway and chemokine genes (Supplementary Figures 9 & 10).

Finally, we ascertained a clinical impact of Shh+ cells in human NSCLC. We prospectively followed all the patients in our study and found a statistically significant association between the percentage of Shh+ cells and the time-to-progression (TTP) for stage I NSCLC. Median TTP was 11.8 months in patients whose tumors showed a percentage of Shh+ cells of more than 0.10%, versus ‘non reached’ in those whose tumors showed a percentage of Shh+ cells of less than 0.10% (p=0.004, Figure 6E). For stage I NSCLC, patients with tumor relapse within 12 months after surgery had a higher percentage of Shh+ cells in their tumors than those without tumor relapse within 12 months of surgery [median: 0.19% (IQR 0.14-0.36) versus 0.05% (IQR 0.02-0.09), respectively, p=0.01; analysis based on 22 stage I NSCLC with follow-up > 12 months after surgery] (Figure 6F).

## DISCUSSION

In this study, we describe a new *in vitro* and *in vivo* lung CSC marker - the membranous full-length Shh protein. CSCs identified by this marker are resistant to systemic chemotherapy but sensitive to GDC0449, a Smoothened inhibitor administered alone or in combination with systemic chemotherapy.

The existence of CSCs has been proven in several malignancies including lung cancer [24, 25]. They are believed to be responsible for tumor initiation and development, as well as resistance to chemotherapy, and thus could be responsible for rapid tumor recurrence or relapse after cancer treatment. Several CSC markers have been described for CSC characterization and isolation such as CD133 [25, 26], CD44 [27, 28], ABCG2 [29, 30] and aldehyde dehydrogenase (ALDH) [31]. However, the specificity of these markers is poor [32–34]. Meng *et al.* [32] have shown that CD133 is not usable for isolation of CSCs in NSCLC because some CD133- cells in NSCLC cell lines presented CSC features (colony formation, self-renewal, proliferation, differentiation and chemoresistance). This marker is probably more specific in SCLC than in NSCLC [33]. Also, the use of ALDH activity is limited in practice because it lacks specificity, as pneumocytes in smoking patients can also have high ALDH activity [34]. Moreover, the functional role of these markers in CSCs remains unclear. Our microarray results in A549 and fresh tumor sorted cells indicate great heterogeneity in the expression of the conventional CSC markers in Shh+ cells.

Although the association of the Shh pathway and CSCs has already been studied in multiple solid tumors [19–22, 35], our study demonstrates that Shh+ cells are CSCs in NSCLC. Whereas during biological development the full-length protein is truncated in the cytosol and only the N-terminal fragment has biological activity, we describe here for the first time the presence and localization of the full-length protein on the membrane of CSCs. The biological activity of full-length Shh protein has already been suggested, but only in *in vitro* studies under physiological conditions [36], and never in cancer models. Shh+ cells exert a paracrine effect on other tumor cells, initiating induction of proliferation and migration signals. IF staining showed a diffuse Shh staining pattern and flow cytometry on permeabilized cells revealed that more than 70% were Shh-positive cells. However, only Shh+ cells are Shh-producing cells, as demonstrated by our ddPCR results on sorted cells, and by the WB results on the supernatant from sorted cells. We speculate that Shh- cells receive the Shh protein secreted by Shh+ cells and internalize the protein immediately after receptor binding, as has been described [37, 38], thereby explaining the cytosolic staining for Shh in permeabilized cells.

Our results also highlight the important role CSCs play in chemo-resistance exhibited by NSCLC cell lines, *in vivo* models (xenografts), and also in patient prognosis (high percentage of Shh+ cells correlated with progressive metastase and responsive lung tumor had low percentage of Shh+ cells). We demonstrate that lung CSCs are sensitive to targeted therapy. Our results show a substantial and significant impact of targeting these CSCs in order to improve clinical outcome of patients treated for lung cancer. Shh+ cells disappeared completely after *in vivo* treatment with GDC0449, and when GDC0449 was combined with chemotherapy, the percentage of Shh+ cells decreased significantly, as assessed via flow cytometry. Previously, GDC0449 was suggested to be effective on lung CSCs, but only *in vitro* and on a very limited number of cell lines [39, 40]. We previously demonstrated that the Shh pathway is overexpressed in chemo-refractory advanced NSCLC treated with platinum-based chemotherapy, and that inhibition of the Shh pathway with GDC0449 had a synergistic effect with cisplatin *in vitro* in the most chemo-resistant NSCLC cell lines [41].

Only a few clinical studies have tested Smoothened inhibitors in lung cancer. The ECOG-ACRIN phase II trial (E1508) tested standard chemotherapy (cisplatin/etoposide) with or without vismodegib as the first line of treatment for advanced-stage SCLC [42]. No effect in terms of response rate, progression-free (PFS) or overall survival (OS) was observed. A recent phase I trial of the Smoothened antagonist (sonidegib) in combination with cisplatin/etoposide for advanced SCLC in 15 patients had a response rate of 79%, which is similar to the rate observed in SCLC patients treated with standard chemotherapy alone; but one patient in this group of 15 who was found to have a *SOX2* gene amplification remained progression-free on maintenance with sonidegib after 27 months [43]. For NSCLC, no published clinical trial has reported tests with Shh pathway inhibitors so far.

In conclusion, we describe here for the first time a unique and unprecedented CSC marker—the full-length Sonic Hedgehog protein—that is present both *in vitro* and *in vivo*. Moreover, unlike other CSC markers, the Shh protein has paracrine and autocrine functions, and is responsible for CSC maintenance, tumor proliferation and resistance to chemotherapy. The presence and role of the full-length Shh protein may lead the way to Shh-targeted therapies. Our results indicate the importance of developing combination treatment strategies for NSCLC. Although systemic chemotherapy is effective at targeting the Shh- cancer cells, Shh-targeted therapies could induce prolonged responses and survival as a result of CSC inhibition.

## MATERIALS AND METHODS

### NSCLC cell lines and culture conditions

All 12 NSCLC cell lines (A549, H322, H441, H460, H522, H838, H1650, H1975, H2228, HCC2935, H1703, H2170) were purchased from American Type Culture Collection (ATCC). Cells were cultured in RPMI 1640, supplemented with 10% fetal bovine serum and 2% antibiotics (Penicillin-Streptomycin). Cells were collected after trypsinization and resuspended in PBS for further analysis. For serum-free medium culture conditions, sorted cells were seeded in non-tissue culture 96-well plates (500 cells per well) and cultured in DMEM-F12 medium (Corning Cellgro), supplemented with basic fibroblast growth factor (bFGF, Invitrogen, 10 ng/ml), epidermal growth factor (EGF, Invitrogen, 20 ng/ml) and insulin (Sigma, 5 μg/ml). Fresh medium with growth factors and insulin was added every 48h. Spheroid formation (appearance of floating cell aggregates) was monitored daily.

### Transfection assays

For transfection assays, we used A549 and H838 cell lines. Transient transfection of the Shh gene was performed with a pCMV-Shh plasmid (Origene). For stable transfected cell lines, we used N-term peptide hemagglutinin (HA)-tagged Shh (1-197aa), C-term peptide FLAG-tagged Shh (198-462aa), double-tagged wild type Shh (N-HA and C-FLAG) and double-tagged cleavage mutant Shh C198A (N-HA and C-FLAG), all in pCMV-Pig vectors. Transfection assays were performed with Lipofectamine 2000 (Life Technologies), according to the manufacturer’s instructions.

### Fresh human tumor samples

Fresh tumor samples were collected directly in the operating room (Surgery Department, University of California, San Francisco) when there was signed pre-operative consent from the patient. Samples were then processed on the same day. Cell dissociation was performed with collagenase type IV (2 mg/ml, 30 min, Sigma), then completed with mechanical dissociation (syringe). Cells were resuspended in PBS for further analysis. Clinical follow-up data were collected prospectively through a database (last time-point: 07/15/2015).

### Shh antibody production

To mount an immune response, Sp2/0-Ag14 mice were injected with both Shh 247-264 AA and Shh 448-462 AA peptides of the human Sonic Hedgehog protein (both are part of the C-term fragment of Shh; C-term: 198-462 AA). The free peptides were used to screen serum-positive samples via ELISA and the best mice were taken to the fusion phase. Approximately 51 antibody clones/sub-clones producing antibodies raised against the C-terminal Shh peptides were screened to identify clones that bind to full-length Shh polypeptide, as follows. First, using flow cytometry, we determined that expression of full-length Shh polypeptide in transfected cells increased the percentage of cells labeled with the Shh antibodies produced by two sub-clones 2D9 and 2G4 as compared to the percentage of untransfected cells labeled by the same antibodies. Next, these antibodies produced by the two sub-clones (2G4, 2D9) were screened by the Shh protein expressed endogenously and/or exogenously in cells, as determined by Western blotting. Once 2G4 and 2D9 were chosen, a large-scale purification of the antibodies ensued and purified antibodies were used for *in vitro* and *in vivo* experiments.

### Flow cytometry and FACS

We used the mouse Shh-C-terminal antibody (mentioned above) for flow cytometry and FACS (1:40). The secondary antibody was a donkey anti-mouse FITC-linked antibody (ab97029, 1:100, Abcam). For cell analysis after treatment assays (chemotherapy or GDC0449), we also used a marker for dead cells (SytoxRed®, Invitrogen). For fresh human samples, we added an anti-CD45 APC-linked antibody (ab28106, 1:100, Abcam), and a marker for dead cells (SytoxRed®, Invitrogen). For staining of stable transfected cell lines, we used a mouse anti-HA antibody (Abcam, 1:100) and a rabbit anti-FLAG antibody (Cell Signaling; 1:400). Corresponding secondary antibodies were donkey anti-mouse AlexaFluor647 (Invitrogen, 1:1 000) and donkey anti-rabbit AlexFluor594 (Invitrogen, 1:1 000). Flow cytometry analyses were performed on an AccuriC6 flow cytometer (BD Biosciences), and FACS on a FACSAria II (BD Biosciences). Flow analyses were carried out with at least 200,000 cells, and each test was performed in triplicate. For all experiments, we used a negative control of cells processed without primary antibody. Sorted cells (Shh+ and Shh- cells) were collected in fresh media, and seeded in culture plates or frozen at -80°C for further analyses.

### Immunofluorescence

Cells were fixed in 70% ice-cold methanol for 20 min and blocked in 5% BSA for 1 hour at room temp. For staining of commercial NSCLC cell lines, we used our mouse Shh-C-terminal antibody (mentioned above) as the primary antibody and donkey anti-mouse FITC-linked antibody (ab97029, 1:100, Abcam) as the corresponding secondary antibody. For staining of non-permeabilized cells, Shh-sorted A549 cells were fixed in 4% paraformaldehyde for 20 minutes and blocked in 5% BSA for 1-2 hours at room temperature. Rabbit anti-Shh (Abcam, 1:200) and lipophilic/membrane-specific Vybrant® CM-DiI Cell-Labeling Solution (ThermoFisher, 1:200) were used according to the manufacturer’s recommendations. A FITC-conjugated anti-rabbit secondary antibody (Abcam, 1:200) and VECTASHIELD DAPI (Vector Laboratories) mounting medium were used to stain cells prior to imaging. For staining of transfected cell lines, we used mouse anti-HA antibody (Abcam, 1:100) and rabbit anti-FLAG antibody (Cell Signaling; 1:400). Corresponding secondary antibodies were donkey anti-mouse AlexaFluor647 (Invitrogen, 1:1,000) and donkey anti-rabbit AlexFluor594 (Invitrogen, 1:1,000) antibodies. Three representative images per well were captured using an LSM 780 confocal microscope at 6300X. Background was subtracted by comparing images only incubated with the secondary antibodies and analyzed using Fiji software. The experiment was performed three separate times and representative images are presented.

### Proliferation and migration assays

MTS proliferation assays were performed (CellTiterGlo 96, Promega) according to the manufacturer’s instructions. The IC_50_ for cisplatin and docetaxel was calculated for each cell line at 72 hours in triplicates.

Wound healing assays were performed to study cell migration. Monolayers of cells were cultured, scratched, and treated with media containing proteins and/or drugs at different concentrations: Shh protein, Shh+ cell culture supernatant, DMSO, and GDC0449. Data was recorded at 0, 24, 72 and 96 hours after treatment.

### Drugs

Cisplatin was purchased from Sigma and reconstituted in PBS (2 g/L). Docetaxel was purchased from Tocris Bioscience and reconstituted in DMSO (5 g/L stock dilution at -20°C; further dilution in fresh culture medium) for *in vitro* assays, or reconstituted in ethanol/polysorbate 80 (1:1; 5 g/L stock dilution at -20°C; further dilution in PBS) for *in vivo* assays. GDC0449 was purchased from Selleck Chemicals and reconstituted in DMSO (30 mM stock dilution). Shh human recombinant protein was obtained from eBioscience and used at 1,200 ng/mL.

### Western blot

Proteins were extracted with M-PER Mammalian Protein Extraction Reagent (Thermo Scientific), according to manufacturer’s instructions. Western blots were then processed following standard protocols. Western blot for membrane proteins was processed using the Mem-PER™ Plus Membrane Protein Extraction Kit (Thermo Scientific). We used a rabbit anti-C-terminal Shh antibody (ab53281, Abcam, 1:1 000). As a secondary antibody, we used a donkey anti-rabbit HRP-conjugated antibody (ab16284, Abcam, 1:1 000). For immunoblotting of supernatants, Shh-sorted A549 cells were cultured for 3 days and the supernatants from Shh- and Shh+ cells were concentrated using Centrifugal Filter Units (Millipore) at 14,000g for 10 minutes at 4°C. A Bradford assay was used to quantify concentrated supernatants for Western blot analysis. Sonic hedgehog (Abcam, 1:1,000) and a loading control for secreted proteins, MMP2 (OneWorldLab, 1:1,000) antibodies were used to probe blots.

### Quantitative RT-PCR and ddPCR

RNA from unsorted cells was extracted with Qiagen’s RNeasy Mini kit (Qiagen) according to the manufacturer’s instructions. RNA from sorted cells was extracted with an Arcturus PicoPure RNA Isolation Kit (Life Technologies). cDNA was then synthesized with an iScript cDNA Synthesis Kit (Bio-Rad) according to the manufacturer’s instructions. Commercial primers for the *Shh* gene were obtained from Origene. Quantitative RT-PCR (qRT-PCR) was performed on an Applied Biosystems 7900HT Fast Real-Time PCR System, in triplicate for each sample. Gene expression analysis was calculated with the delta-delta CT method normalized to an endogenous control (*18S* gene). For ddPCR, droplet creation, PCR and data analysis were performed according to the manufacturer’s instructions (BioRad, QX100 ddPCR System, Quantasoft software). Results obtained from ddPCR were expressed as FAM concentrations (copies/μl).

### Microarray

Total RNA (about 25 ng) was amplified into cRNA and made into cDNA using the Ambion WT Expression Kit (Life Technologies) or the Ovation Pico WTA System V2 kit (NuGen). The cDNA (5.5 μg for the Ambion WT Expression kit, 2.5 ug for the Ovation Pico WTA System V2 kit) was then fragmented using the Affymetrix GeneChip WT Terminal Labeling kit (Affymetrix, Santa Clara, CA, USA) and confirmed by running 1 μl of each sample on the Agilent Bioanalyzer using the RNA 6,000 kit (Agilent Technologies, Santa Clara, CA, USA). The fragmented cDNA was labeled using the Affymetrix GeneChip WT Terminal Labeling kit and added into the hybridization cocktail that was prepared according to the protocol included in the Affymetrix GeneTitan Hybridization Wash and Stain kit (Affymetrix). The samples were finally loaded into the Affymetrix GeneTitan MC for hybridization, washing and scanning.

### Mice

For xenograft formation, 5-10 week-old female nude mice, were injected subcutaneously (SC) with 10 million A549 cells in the dorsal area in a volume of 100 μl. For SC inoculation of sorted cells, 1,500 cells of A549 Shh+ or Shh- cells were injected in a volume of 150 μl. For intravenous injection of sorted cells, 1,000 cells of A549 Shh+ or Shh- cells were injected in the tail vein. For the *in vivo* treatment assay, after xenograft formation, animals were injected intravenously with cisplatin (10 mg/kg, weekly), docetaxel (10 mg/kg, twice a week) or vehicle, and intraperitoneally with GDC0449 (20 mg/kg, daily) or vehicle. Each group consisted of 4-5 mice. Tumor size was determined twice a week, and tumor volumes were calculated using width (*x*) and length (*y*) (*x*^*2*^*y/2*, where *x<y*). Tumors were collected after mice were euthanized, and cell dissociation was performed in the same way as for fresh human samples as described above. Flow cytometry and FACS were performed as described above. All animals were cared for in accordance with guidelines from the Institutional Animal Care and Use Committee at the University of California, San Francisco (UCSF).

### Statistical analyses

Distribution of variables was analyzed by the Shapiro-Wilk test. For normally distributed variables, results were expressed as mean (±SD), and comparison between 2 populations was performed with Student’s *t*-test. For variables that were not normally distributed, results were expressed as a median (interquartile range IQR), and comparison between 2 populations was performed with a non-parametric Mann-Whitney test. For qRT-PCR data analyses, we used a Student’s *t*-test. For ddPCR results, we used Poisson law, with Poisson confidence intervals. Time-to-progression was calculated with the Kaplan-Meyer log-rank test. For each test, results were considered as significant if p<0.05. Statistical analyses were performed using Xlstat 2.01 software (Addinsoft).

## SUPPLEMENTARY MATERIALS FIGURES AND TABLE


