# Supplementary material for: Membrane-bound full-length Sonic Hedgehog identifies cancer stem cells in human non-small cell lung cancer
Source: Oncotarget. 2017 Oct 10;8(61):103744–57. doi: 10.18632/oncotarget.21781 (PMC5732763; doi:10.18632/oncotarget.21781)
Supplement: Supplementary file 1 [file oncotarget-08-103744-s001.pdf]

Membrane-bound full-length Sonic Hedgehog identifies cancer stem cells in human non-small cell lung cancer

SUPPLEMENTARY MATERIALS

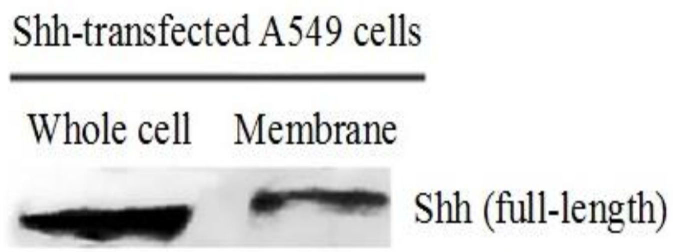

Supplementary Figure 1: Immunoblot of A549 cells transiently transfected with wild-type *Shh* and probed for the Sonic Hedgehog (Shh) protein in whole cell and membrane extracts.

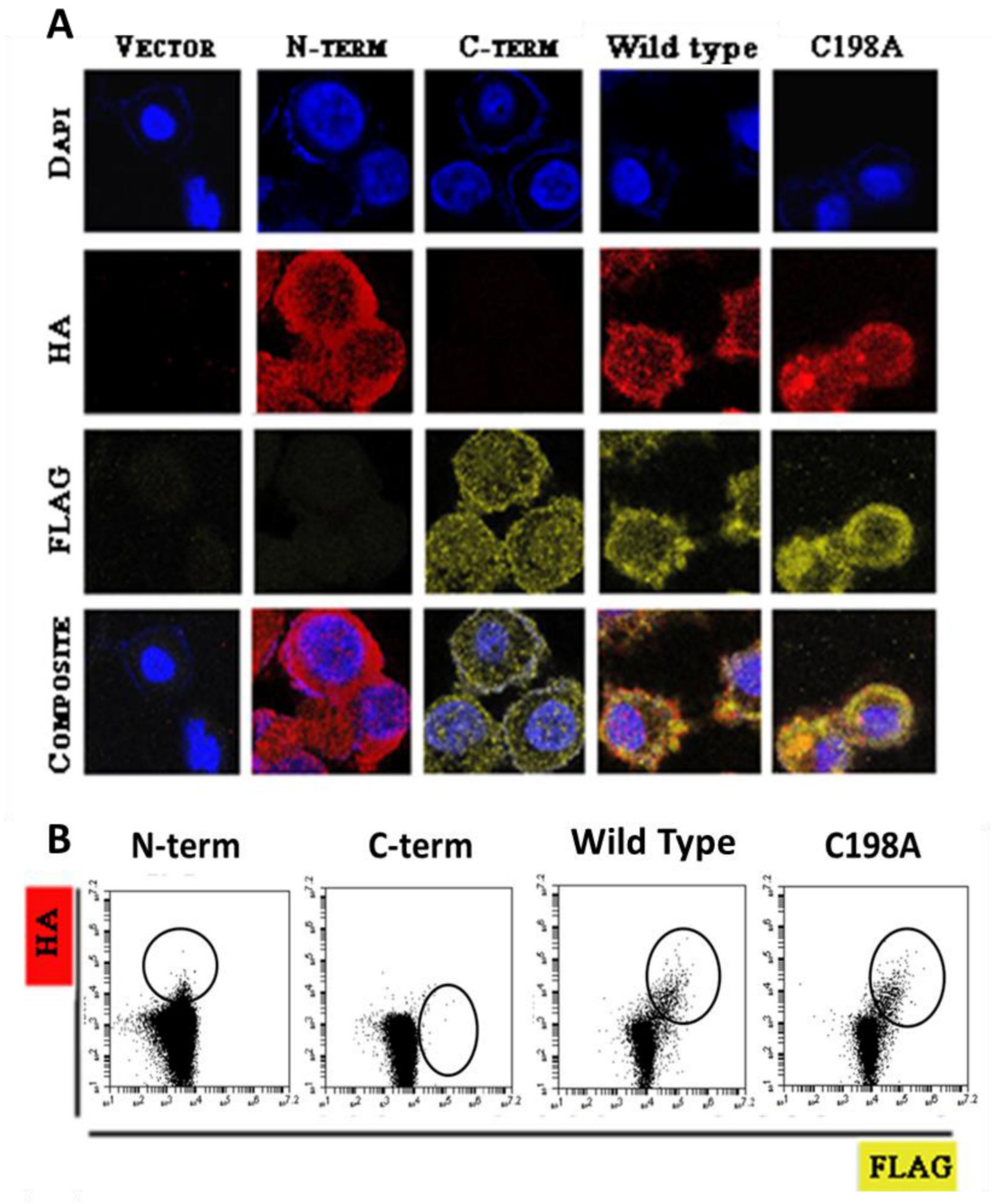

Supplementary Figure 2: (A) Immunofluorescence analysis of A549 cells showing cytosolic and membrane staining of N-term, C-term, wild-type Shh and C198A Shh constructs probed for the presence of HA (red) and FLAG (yellow). **(B)** Flow analysis showing positive double-staining in H838 cells for HA and FLAG in cells bearing wild-type and C198A constructs and single staining for N-term and C-term.

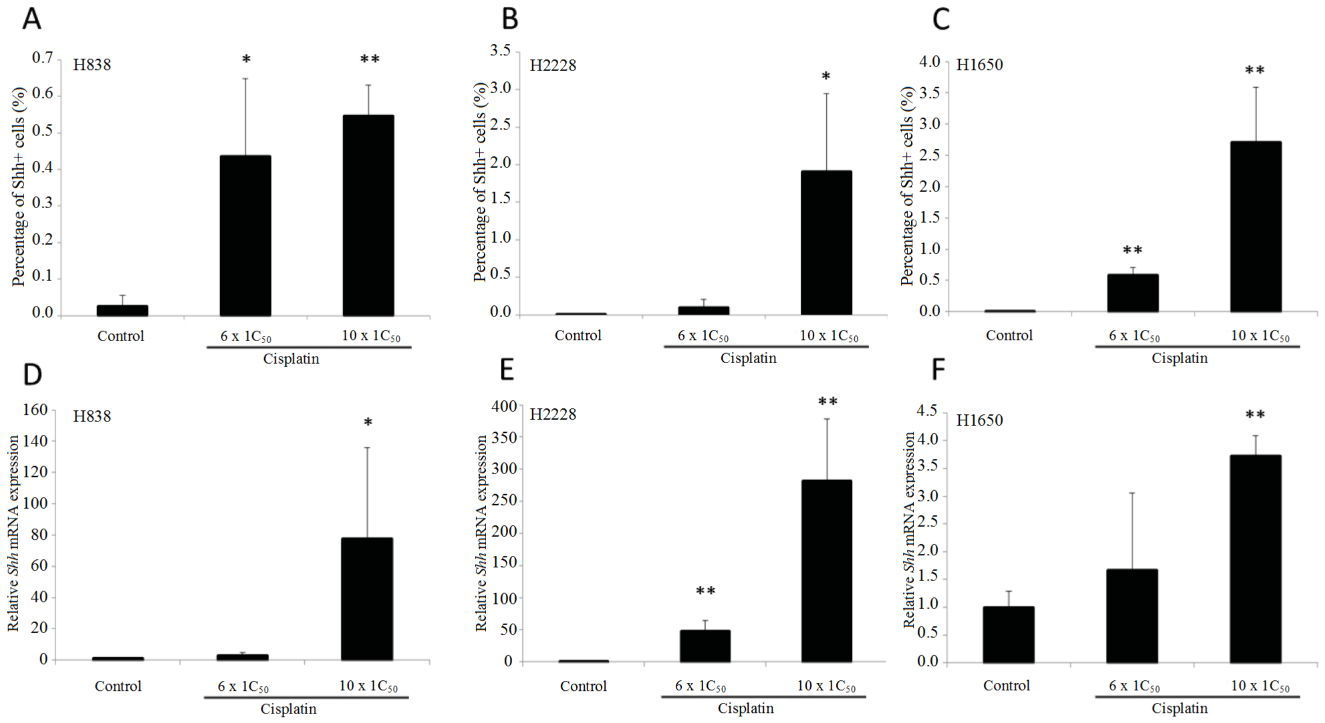

Supplementary Figure 3: (A-C) Percentage of Shh+ cells (%) assessed by flow cytometry in H838 (A), H2228 (B) and H1650 (C) cells treated with 2 doses of cisplatin (6-fold and 10-fold IC_{50}, 72h). **(D-F)** *Shh* gene expression levels in H838 (D), H2228 (E) and H1650 (F) cells treated with 2 doses of cisplatin (6-fold and 10-fold IC_{50}, 72h) analyzed by qRT-PCR (normalized to PBS-treated cells).

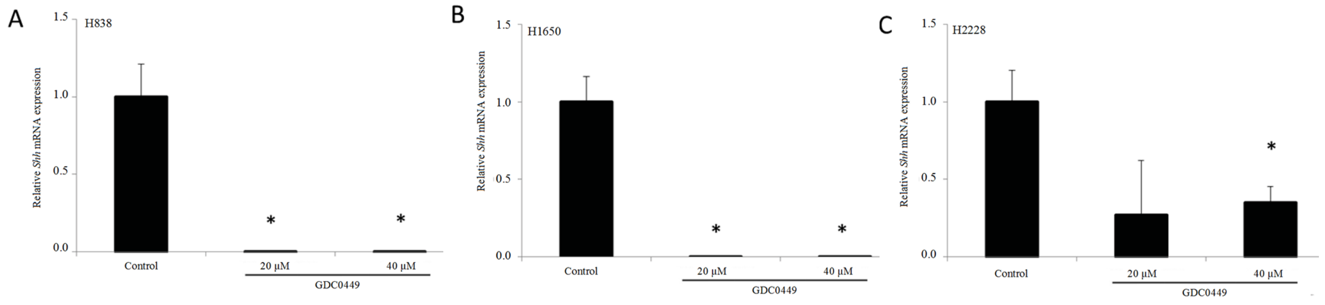

Supplementary Figure 4: *Shh* gene expression levels (qRT-PCR) in H838 **(A)**, H1650 **(B)** and H2228 **(C)** treated with GDC0449 (20 µM and 40 µM, 72h) (normalized to DMSO-treated cells).

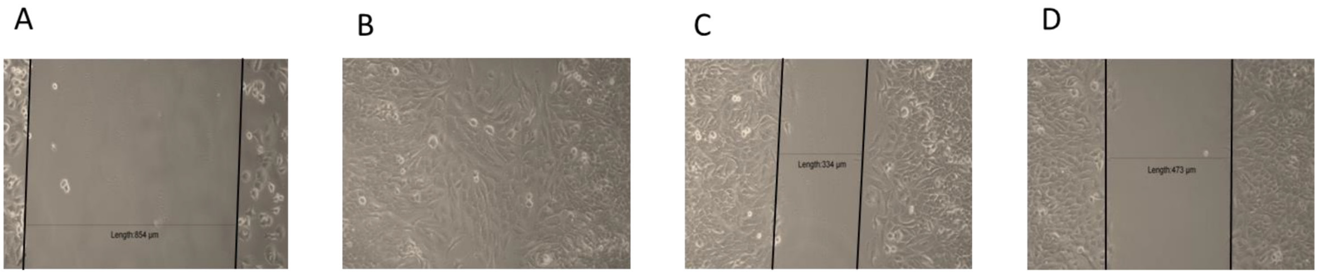

Supplementary Figure 5: Migration/wound healing assay performed on A549 cells at d0 **(A)** and d3 **(B)** DMSO; **(C)** GDC0449 20 μM; **(D)** GDC0449 40 μM.

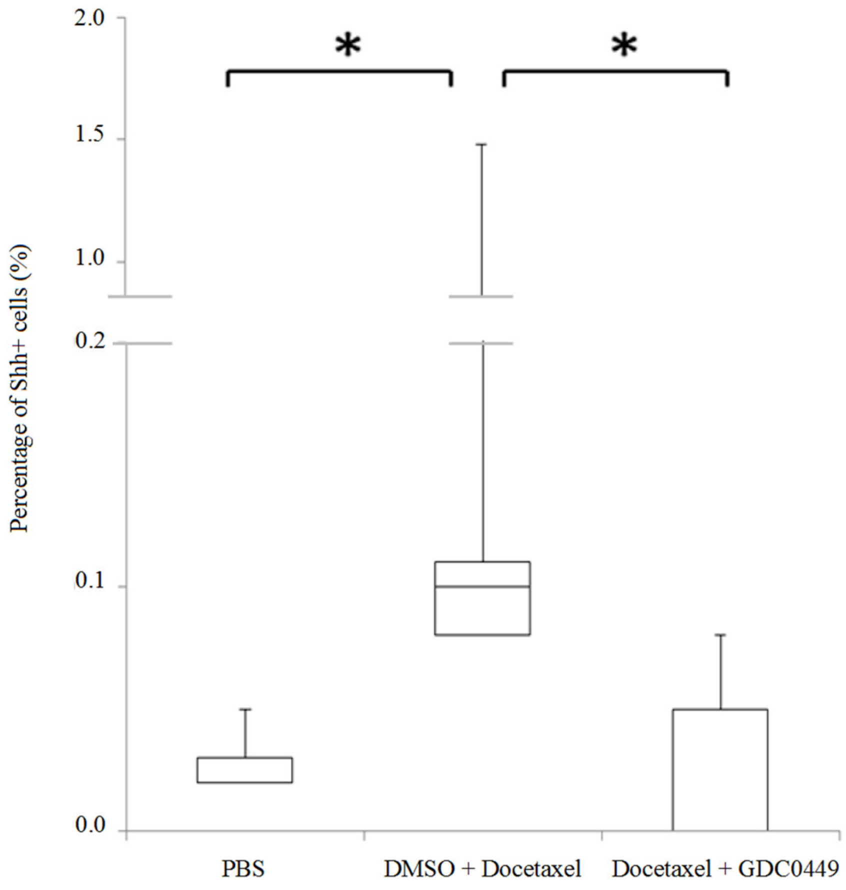

Supplementary Figure 6: Percentage of Shh+ cells (%) at d21 in A549 xenografts (nude mice) treated with PBS or docetaxel (10 mg/kg, twice a week) plus vehicle or docetaxel (10 mg/kg, twice a week) and GDC0449 (20 mg/kg, IP daily). *p<0.05.

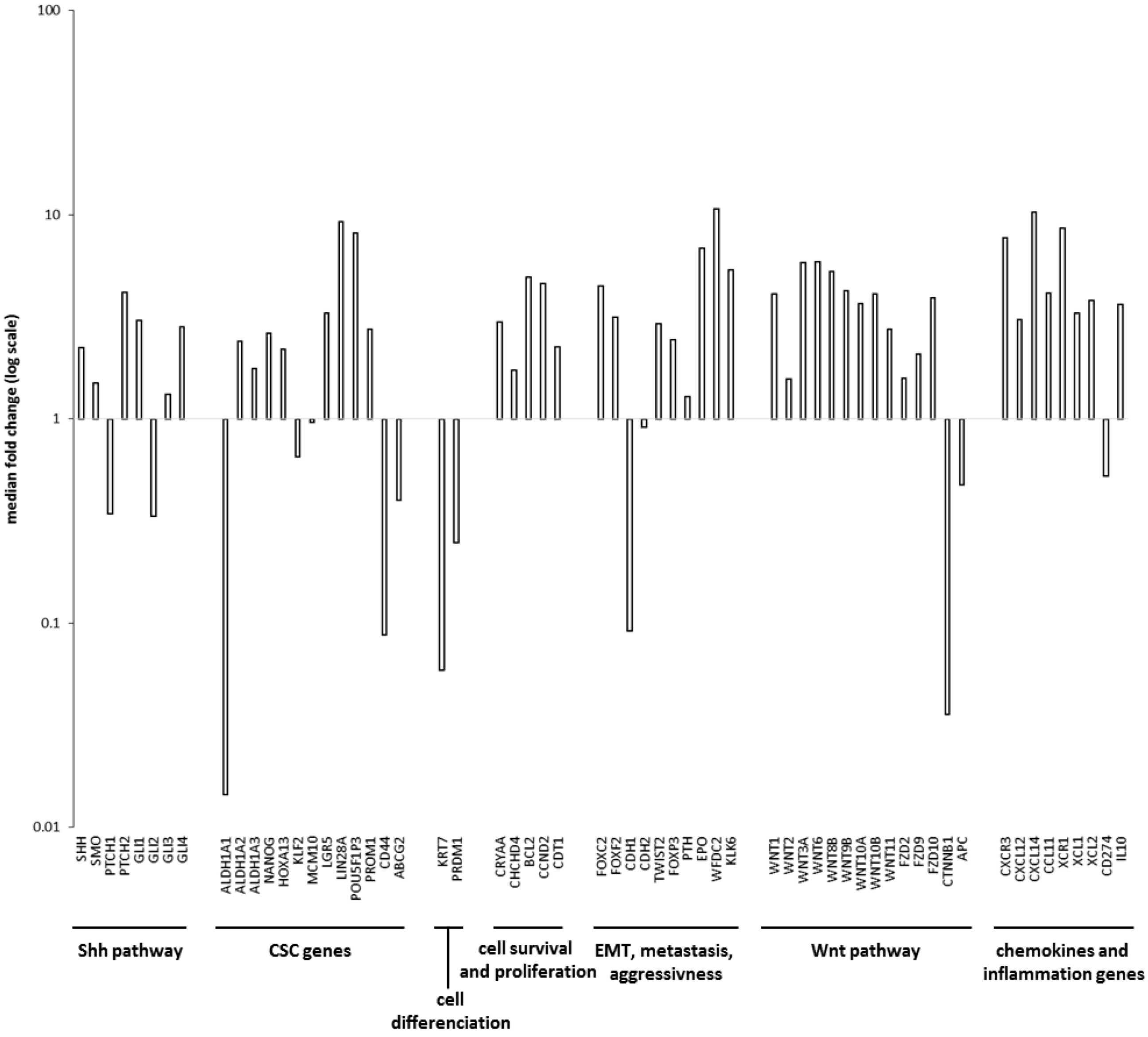

Supplementary Figure 7: Microarray gene expression analysis on A549 Shh+ cells (normalized to Shh- cells, log-scale). CSC: cancer stem cell. EMT: epithelial-mesenchymal transition.

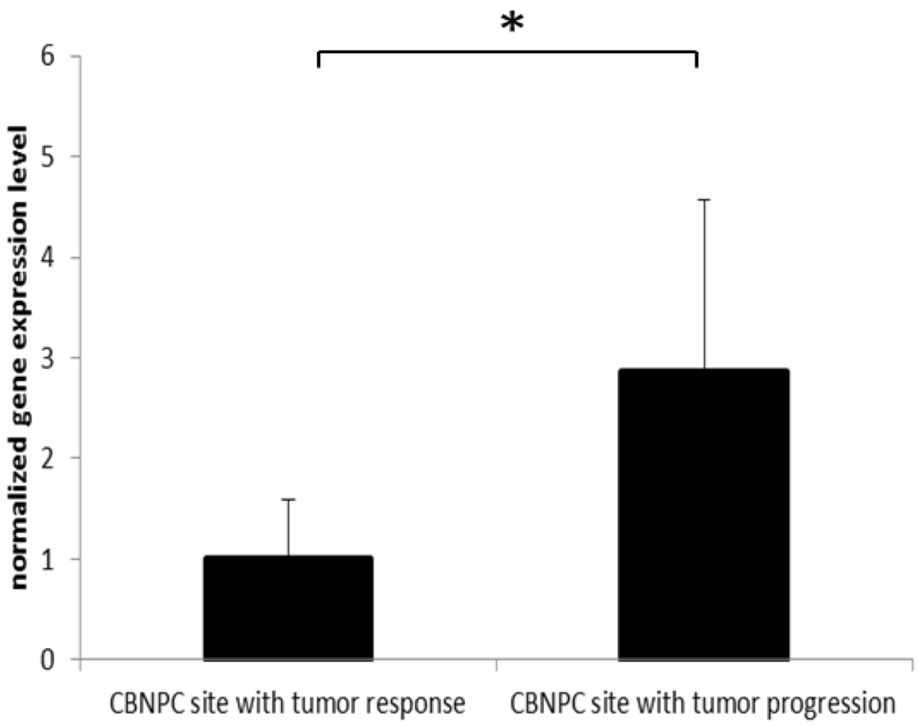

Supplementary Figure 8: *Shh* gene expression level (qRT-PCR) in the primary lung adenocarcinoma with tumor response after chemotherapy, and the corresponding adrenal metastasis with tumor progression after chemotherapy in the same patient. *$p<0.01$.

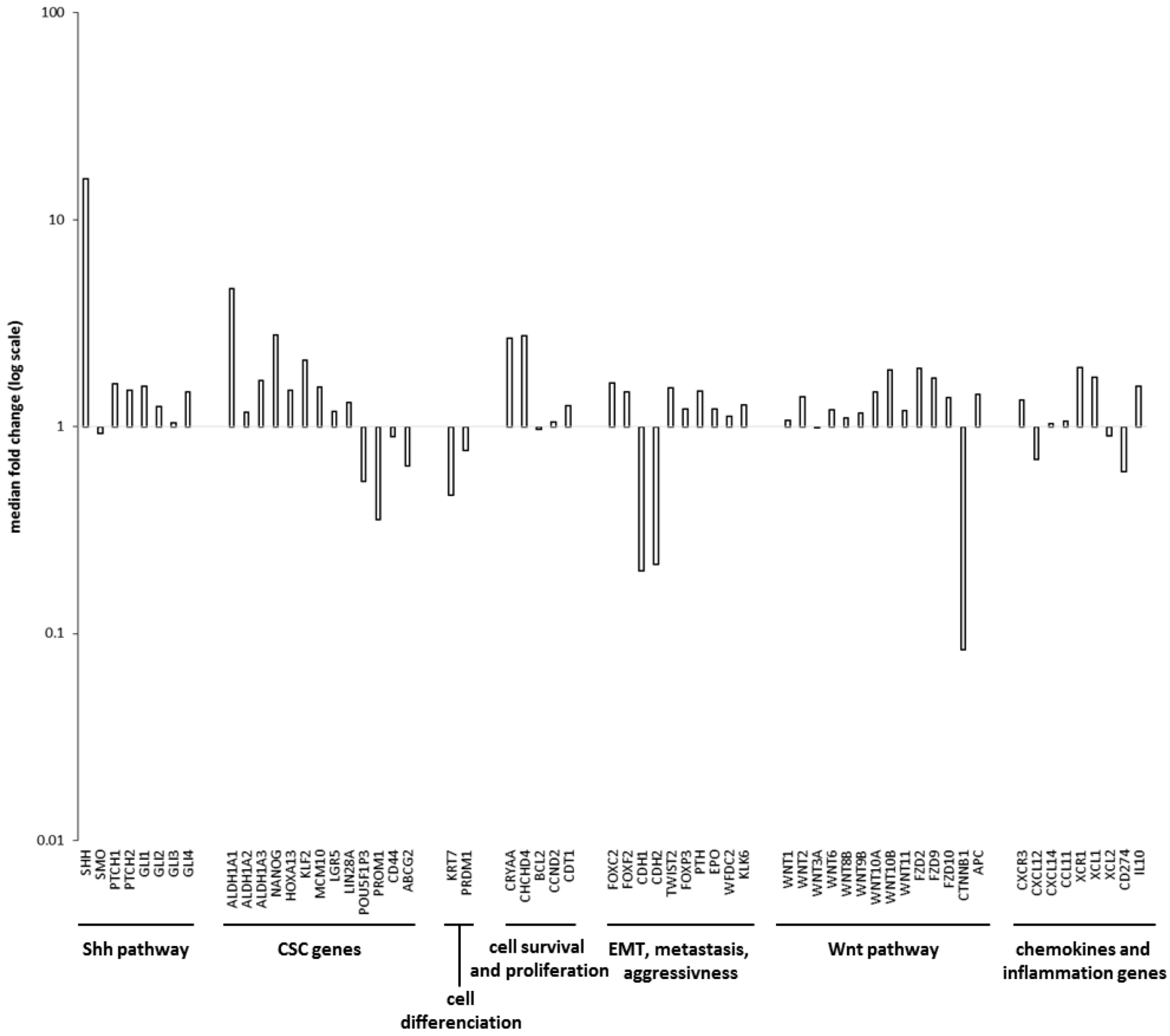

Supplementary Figure 9: Microarray gene expression analysis on Shh+ cells from fresh lung adenocarcinoma (normalized to Shh- cells; log-scale; Percentage of Shh+ cells at 0.69% assessed via flow cytometry). CSC: cancer stem cell. EMT: epithelial-mesenchymal transition.

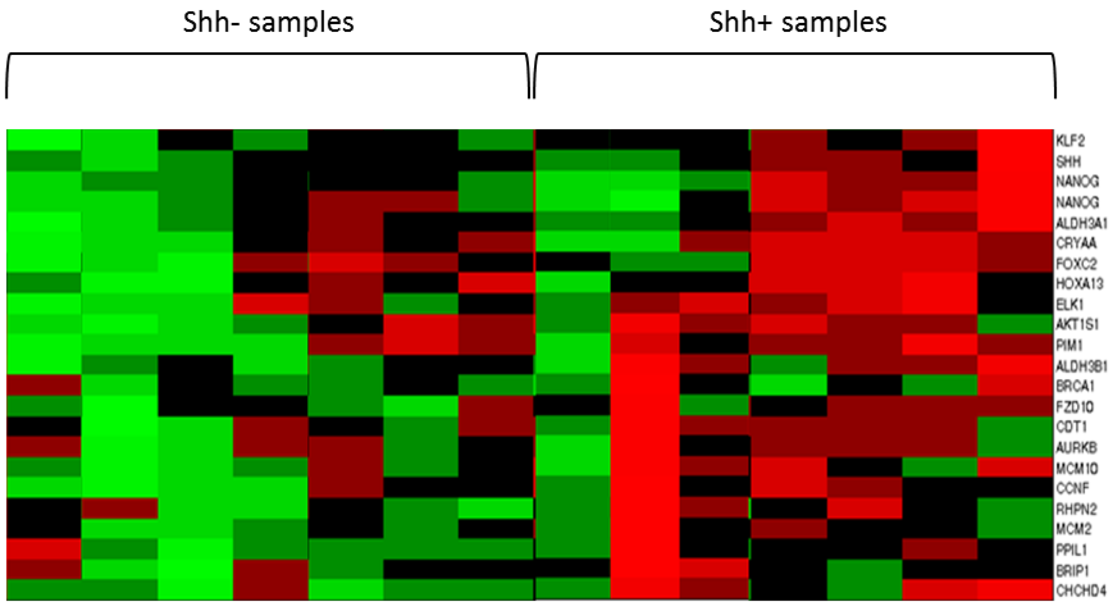

Supplementary Figure 10: Heat map of microarray gene expression analysis in Shh+ and Shh- cells isolated from human fresh lung tumor samples (n=7). Genes analyzed were from the Shh pathway (*Shh*), Wnt pathway (*Fzd10*), those involved with CSC features (*NANOG, HOXA13, KLF2, ALDH3A1, ALDH3B1*), cell proliferation (*CRYAA, CDT1, AURKB, MCM2, MCM10, CCNF, RHPN2, AKT1S1, PIM1*), EMT (*FOXC2*), hypoxia and tumor proliferation (*CHCHD4*), and chemoresistance (*BRCA1, BRIP1, ELK1*).

Supplementary Table 1: Shh rate (%) evaluated by flow cytometry in various fresh tumor samples

Pathological type of lung metastases	n	Shh rate (%)
Malignant pleural mesothelioma	7	0.07 (IQR 0.02-0.13)
Melanoma	5	0.10 (IQR 0-0.42)
Sarcoma	3	0.02 (IQR 0.02-0.04)
Prostatic carcinoma	1	0.07
Renal carcinoma	1	0.06
Breast carcinoma	1	0.01
Colorectal carcinoma	1	0.01
Hepatocarcinoma	1	0.01

Shh rate expressed as median (IQR) if several samples were tested for each pathological subtype.
